# Dynamics of submicroscopic and microscopic asymptomatic malaria infection and associated factors: A longitudinal study in South Benin

**DOI:** 10.1371/journal.pone.0311217

**Published:** 2024-12-12

**Authors:** Rodeline Telfils, Akpéyédjé Yannelle Dossou, Armel Djènontin, Elisée Adimi, Romuald Akoho, Justine Bailly, Aziz Bouraïma, Déborah Matondo, Yolande Sissinto-Savi de Tove, Dismand Houinato, Achille Massougbodji, Célia Dechavanne, Gilles Cottrell

**Affiliations:** 1 IRD, MERIT, Université Paris Cité, Paris, France; 2 Institut de Recherche Clinique du Bénin/IRCB, Abomey-Calavi, Bénin; 3 Centre de Recherche Entomologique de Cotonou (CREC), Cotonou (Bénin) and Centre de Recherche pour la Lutte Contre les Maladies Infectieuses Tropicales (CReMIT), Université d’Abomey-Calavi (UAC), Cotonou, Bénin; 4 Centre d’Étude et de Recherche sur le Paludisme Associé à la Grossesse et à l’Enfance (CERPAGE), Faculté des Sciences de la Santé, Cotonou, Bénin; 5 Faculté des Sciences de la Santé, Université d’Abomey-Calavi, Cotonou, Bénin; University of Cape Coast College of Health and Allied Sciences, GHANA

## Abstract

**Introduction:**

Asymptomatic malaria infection is now recognized as a potential threat to malaria control. However, its prevalence and its dynamics are poorly documented especially in a perennial context of high seasonal transmission. A longitudinal study was conducted in southern Benin to investigate the dynamics of asymptomatic malaria infection and to identify factors influencing it.

**Methods:**

A cohort of 377 participants was recruited, stratified into three age groups (under 5 years, between 5 and 15 years, over 15 years). After inclusion, two visits were made one-month apart between August and November 2021. Malaria infection was diagnosed by microscopy and PCR and questionnaires were administered to the participants. The dynamics of malaria infection, both submicroscopic (positive PCR / negative blood smear) and microscopic (positive blood smear), and related factors were determined using a mixed ordinal polytomous regression model and a multistate Markov model.

**Results:**

The human infectious reservoir consisted primarily of asymptomatic submicroscopic infections (289/512 (56.4%)), followed by asymptomatic microscopic infections (182/512 (35.5%)) and symptomatic infections (41/512 (8%)). The prevalence of asymptomatic infection was highly related to age-group (5–15 years: OR: 4 .12 [2.55–6.67] and > 15 years OR: 2.80 [1.73–4.54] compared to the under 5 years old group). The children under 15 years with asymptomatic infection had the highest risk of becoming symptomatic. The mean duration of asymptomatic infections in 5–15-year-olds was the longest (76.7 days (53.8–109.1)).

**Conclusion:**

This study revealed a persistent asymptomatic malaria reservoir over the follow-up period, with substantial variations between age-groups. These findings are important elements to consider for an optimal deployment of malaria control interventions.

## Introduction

Africa remains the continent with the highest malaria burden, accounting for 94% of cases (233 million), over 90% of which are attributed to *Plasmodium falciparum*, the main cause of malaria-related deaths [1]. In Benin alone, an estimated 4.9 million cases and 10,000 deaths were recorded in 2021 [2]. The World Health Organization (WHO) has prioritized the fight against malaria through the prevention and treatment of symptomatic cases. In most African countries, compliance with WHO recommendations led to a significant 62% reduction in malaria morbidity and mortality between 2000 and 2015 [2]. However, progress has stalled since 2015 and it is now recognized that current control tools will not be sufficient to achieve elimination [3]. There is growing evidence for the need to understand the human reservoir of asymptomatic carriers.

Asymptomatic malaria, is defined as the presence of parasites in people and an absence of malaria-related symptoms (such as fever) [4, 5]. Asymptomatic infections represent the majority of all malaria infections, and can be missed by surveillance strategies based on microscopy or detection of antigens by rapid diagnostic tests (RDT) [6, 7]. Polymerase chain reaction (PCR) has made it possible to detect the so-called submicroscopic infections (negative by microscopy and positive by PCR) which are generally asymptomatic [8–11].

In Malawi in 2015, a cross-sectional study showed that over 88% infected with Plasmodium were asymptomatic, particularly school-age children aged 6–15 and adults [12], who can constitute a reservoir for malaria transmission. Acquired immunity to the disease leads to persistent, asymptomatic, or mildly symptomatic infections [7, 11], that often go undetected. Such infections are less likely to be treated than cases of acute febrile illness, which occur more frequently in children under 5 [5]. Asymptomatic persistent infections have been shown to be mainly responsible for malaria parasite population maintenance between transmission seasons [13] in the Sahelian epidemiological context.

However, the epidemiology, dynamics, and determinants of asymptomatic malaria reservoir are still poorly understood, in areas of high perineal transmission [14]. A longitudinal study conducted between 2017 and 2019 in Uganda showed that 83.6% of the infectious reservoir originated from asymptomatic microscopic infections, 15.6% from asymptomatic submicroscopic infections and only 0.6% from symptomatic infections. This shows the importance of the contribution of the asymptomatic reservoir in malaria transmission [15], including submicroscopic infection, as shown also in Sahelian context [16]. However, the precise contribution of submicroscopic versus microscopic parasitemia to transmission remains uncertain. Although the known major role of individual acquired immunity to control malaria infection, the factors determining why an infection remains submicroscopic or becomes microscopic (eventually symptomatic) remain also unclear.

To assess the dynamics of asymptomatic malaria infections and its related factors in facies of perennial seasonal transmission, we conducted a longitudinal study in a region of intense transmission in Benin.

## Methods

### Study site

This study was conducted in the rural areas of Adjrako in southern Benin (N 06°36′14′′; E02°17′39′′), located in the municipality of ze, which is one of the three municipality in the Allada -Ze- Toffo health zone, in the Atlantic Department. The average annual rainfall is 1257.95 mm, and the average temperature is 27.4°C. The main vectors in the setting are Anopheles gambiae, and Anopheles funestus, according to an entomological survey conducted in concurrently with this study, and the overall entomological inoculation rate (EIR) for the three months of collection (September to November) of 8.7 infecting bites per person [17]. The climate is sub-equatorial, with two rainy seasons extending from April to July and October to November with *Plasmodium falciparum* as the major infecting species in the district. Malaria is perennial and highly endemic in the study area and malaria transmission occurs year-round [18]. The area of Yokpo where Adjroko’village is located has no health facilities and this study allows us to provide access to care for the community.

### Study design

**Fig 1:**
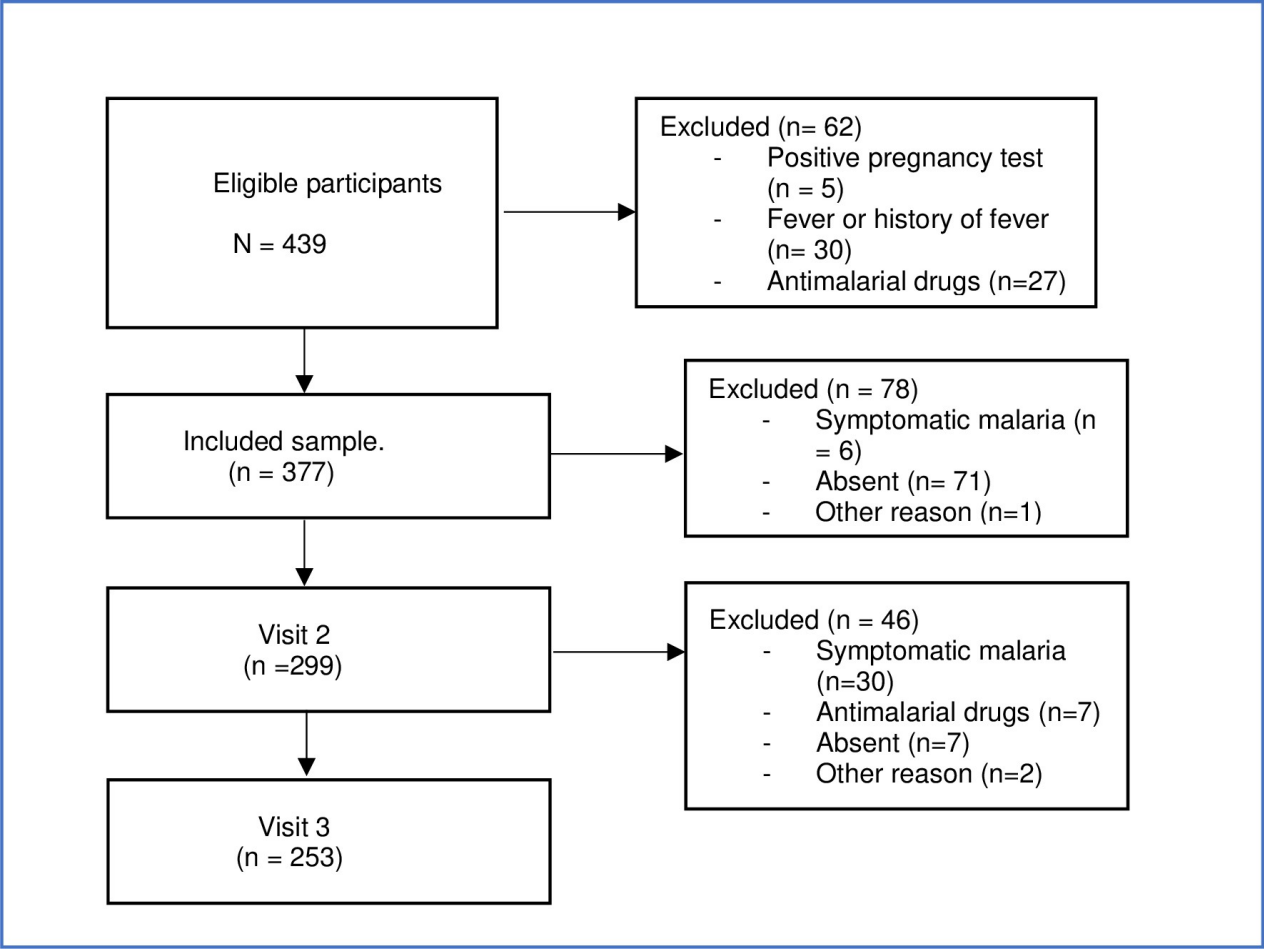
The flow chart of the study.

A cohort was followed-up between August and November 2021. A total of 377 participants were recruited from the general population (Fig 1). The recruitment process consisted in meetings at central assembly areas in Adjrako. During these meetings, the study was presented to community members, along with detailed information on study procedures and conditions for participation. To be included, participants had to be voluntary and asymptomatic. They were stratified into three age groups: under 5, between 5 and 15 and over 15, after the study had been explained to them and they had given their free consent.

Subjects who reported having fever 72 hours before enrolment, those with fever at moment of examination (defined as a body temperature of 37.5°C or higher), a travel notion within the next 3 months, a positive urinary pregnancy test, reported intake of any antimalarial drug within the previous 28 days or chronic illnesses and symptomatic forms of malaria, and children aged under 1 year were excluded.

Temperature, weight, height, urine pregnancy test for women and girls over 16 years of age, were recorded. Symptomatic malaria infection was defined by a positive RDT plus fever (temperature ≥ 37.5°C) or history of fever in the last 72 hours.

Any participants with severe malaria or any diagnosed ailments that could not be treated by study staff were referred to the nearest health facilities. RDT positive and symptomatic individuals received treatment with Artemether-Lumefantrine (AL) (20 mg artemether and 120 mg lumefantrine [Coartem®, Novartis Pharma AG, Basel, Switzerland]) or AL dispersible, twice daily for three consecutive days. Pregnant women were referred to the nearest health center for pregnancy monitoring and malaria chemoprophylaxis.

### Follow-up

At enrolment, between August 16th and September 6th, 2021, demographic and socio-economic characteristics as well as use of mosquito nets and preventive measures were collected using questionnaires. Three separate visits, conducted in groups at community assembly areas in the village, were organized to follow participants for the duration of the study. The first visit was for enrolment. The next visit was conducted between 3 and 5 weeks after inclusion. Finally, the third visit took place between 6 and 9 weeks after inclusion. At enrolment and at each visit peripheral blood was collected on EDTA by nurses to assess malaria infection. One drop of peripheral blood was collected using finger prick to fill HemoCue® microcuvette.

During the follow-up, malaria infections were detected by both thick blood smear (TBS) and PCR. Only symptomatic infections were treated with AL combination therapy according to national recommendations and participants were taken out of the study. Asymptomatic infections were not treated during follow-up, but treatment was given to all positive individuals at the end of follow-up.

In addition to systematic and scheduled visits, the field team, assisted by community health workers, also carried out weekly general health check-ups all along the study to detect any sign of concern that might have arisen between scheduled visits.

### Laboratory procedures

RDT was performed at inclusion or during the follow-up in case of fever, based on the detection of *Pf*HRP2 antigen (Histidine Rich Protein 2) [19].

Microscopic diagnosis was made by thick drop–blood smear. Microscopic parasitaemia was quantified by the standardized Lambaréné approach, which consists in detecting the totality of the blood smear taken from a drop of 10μL-calibrated volume [19–21]. This approach has an estimated detection threshold of 5 parasites/μl and has better sensitivity than the conventional thick smear count [20]. TBS were considered positive if at least one trophozoite was visualized after examination of 1,000 leukocytes. TBS were read by 2 microscopists and discordances between the 2 readings or between microscopy and PCR were confirmed by a third microscopic examination.

Detection of *Plasmodium* infections was also performed using a real-time quantitative polymerase chain reaction (qPCR) targeting the 18S ribosomal deoxyribonucleic acid (rDNA) gene [22, 23]. The primers used for the assay target three independent Plasmodium genes: the P. falciparum cytochrome b mitochondrial (Pf cytb), the P.malariae 18S rRNA sequence (Pm 18s) and the P. ovale dihydrofolate reductase (Po dhfr) genes. The qPCR assay was performed on the Viia7™ Real-Time PCR System (ThermoFisher Scientific, Massachusetts, US) and QuantStudio 5 (Applied Biosystems, Massachusetts,US). The thermal profile for all qPCR was as follows: initialization step at 95°C for 15 min, 40 cycles of 15 s at 95°C, 20 s at 63°C for Pf and Pm, 58°C for Po respectively and 20 s at 72°C min. The reaction wells for the P. falciparum, P. malariae and P. ovale qPCR contained 3 μL of 5X HOT FIREPol EvaGreen Mix Plus (Solis BioDyne, Tartu, Estonia), 0.5 μM of each primer (Pfcytb, Pm18s, and Podhfr) and 2.5 μL DNA for a total of 15 μL. Primers were Supplied by Integrated DNA Technologies.

Hemoglobin levels were measured on the field using the HemoCue® Hb 201+ System. Anaemia was defined as a binary variable by different thresholds according to age and sex, in line with WHO parameters:

- if age is less than 2 years and haemoglobin level is less than 10.5 mg/dl
- if age is between 2 and 12, and haemoglobin level is below 11.5 mg/dl
- If women are aged between 12 and 80, and haemoglobin levels are below 12 mg/dl
- If men are aged between 12 and 80, and haemoglobin levels are below 13 mg/dl.

### Ethical consideration

This study obtained the ethical approval from institutional ethical committee from the Centre de Recherche Entomologique de Cotonou (CREC/CEI-CREC/SA N° 26). Prior to study initiation, a community meeting was held in each hamlet of the village to discuss the study objectives with the communities and community leaders. Written informed consent was obtained from each participant prior to recruitment. All participation was voluntary. Parental or guardian consent was obtained for minors. If a parent or guardian was illiterate, community leaders or health workers helped in familiarizing the parent/guardian with the study.

### Statistical analysis

All data collected during the survey were recorded in electronic forms on smartphones installed with Open Data Kit (ODK) collect. Data analyses were performed using R studio software version 4.2.1 [24].

The overall prevalence of malaria was calculated by dividing the number of positive samples by the total number of blood samples examined by microscopy and PCR. The prevalence of submicroscopic infections was determined as the percentage of PCR-positive samples, while the prevalence of microscopic infections was the percentage of microscopy-positive samples. The contribution of each type of infection (submicroscopic, microscopic and symptomatic) to the total infectious reservoir was assessed by calculating its ratio to overall infections detected.

We performed first an ordinal polytomous mixed model to assess the determinants of malaria infection dynamics. In this model our dependent variable was a time-dependent, ordinal variable with 3 classes (negative, submicroscopic, and microscopic infection), summarizing the malaria infection status of the participants in each visit. At each visit, the malaria infection was defined as negative if all tests (TBS and PCR) were negative; as a submicroscopic infection if the TBS was negative but the PCR was positive; and as a microscopic infection if the TBS was positive.

Explanatory variables were gender, age group (under 5 years, between 5 and 15 years, over 15 years), net ownership, net use the previous night, month of visit, and herbal tea consumption used as malaria prevention.

In a first step, we performed a univariable analysis to assess the association between each explanatory variable and asymptomatic malaria infection. We selected the explanatory variables based on their p-value (p < 0.2) effect size from the univariable step and expertise to be included in the model. Next, we performed a multivariable model that simultaneously included the selected explanatory variables. Age group and month of visit were forced in the model. We performed a Brant test to assess the proportional odds assumption, and the assumption was met [25].

In an ordinal logistic mixed model, the estimated odds ratios according to the covariates cannot be directly interpreted as the comparison of the risk of a submicroscopic (respectively microscopic) infection status versus a negative status. Therefore, we derived from the model the adjusted predicted probabilities (risk) of a malaria infection status and their confidence intervals.

**Fig 2:**
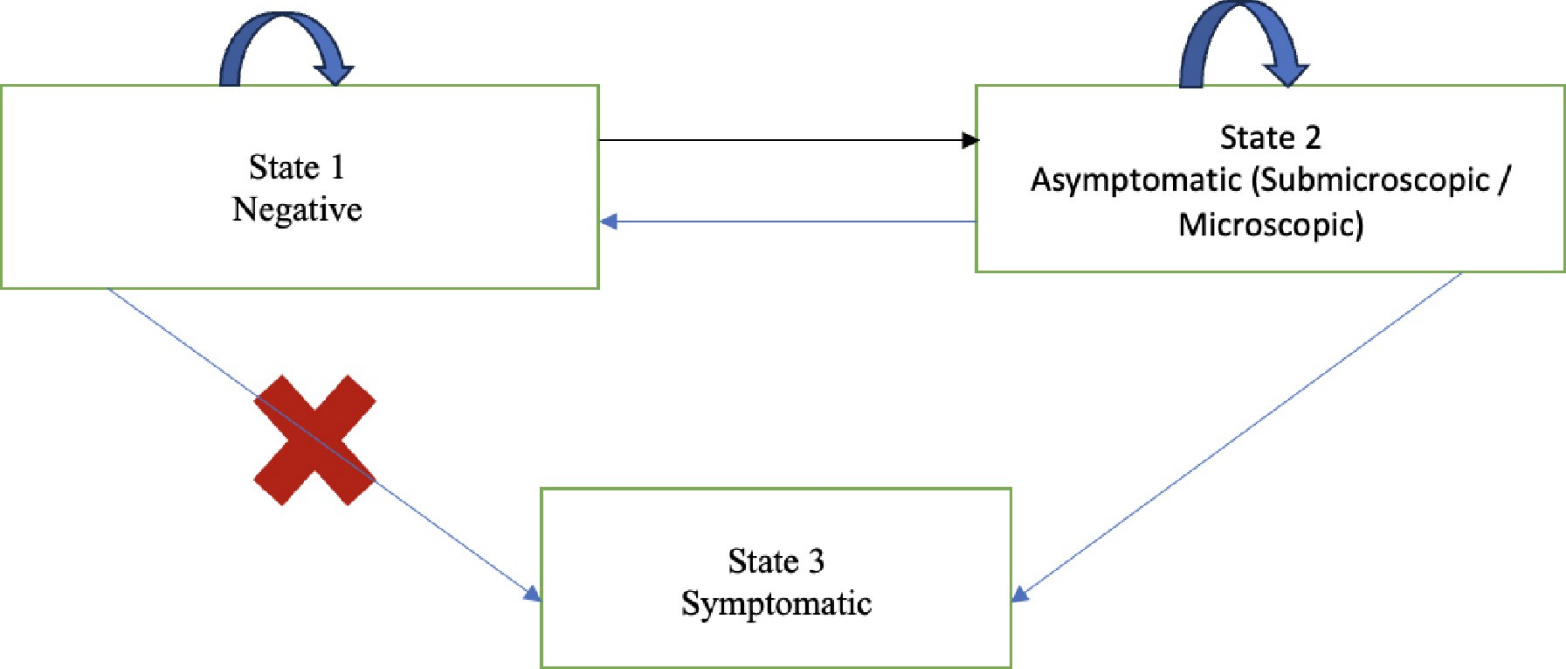
Three states homogenous Markov model.

In a second analysis we built a three-state homogeneous Markov multi-state model to estimate the transition probabilities between the infection status and their corresponding sojourn times. Data were interval-censored, meaning that observations of the different states were made at regular times, but the exact transition times between states were not known. To estimate transition probabilities between states, and sojourn times for the age groups in each state, we used the MSM (Multi-State Models) package developed by Jackson in 2011, integrated into R software [26]. Thus, the three state was either:

1. State 1 (Negative) represented individuals who were not infected with malaria. From this state, individuals could remain negative or move to the asymptomatic state (state 2). there was no direct transition between state 1 and state 3.
2. State 2 (Asymptomatic infection) represented individuals who have been detected to be infected with malaria, whether by microscopy or PCR, without clinical signs. They could return to the negative state, remain in this asymptomatic state, or progress to the symptomatic state (state 3).
3. State 3 (Symptomatic) represented individuals detected to be infected with malaria and exhibiting clinical symptoms such as fever. the symptomatic state was an absorbent state as the participants were treated and taken out of the study. Fig 2 illustrates the 3-state model.

## Results

The overall prevalence of malaria was 53.8% (512/952). The prevalence of submicroscopic infection was 30.4% (289/952), while microscopic infection was 23.4% (223/952). Table 1 presents the general characteristics of the study population. During follow-up, 377 participants were included, stratified into three approximately balanced age groups. Females slightly represented the majority, with a sex ratio (F/M) of 1.27. Most participants owned a mosquito net and slept under one.

**Table 1:** General characteristics of the population by age group at inclusion.

Characteristics	Less than 5 years (n = 122)	5–15 years (n = 123)	More than 15 years (n = 132)
**Gender**			
Male	59 (48.3%)	63 (51.2%)	44 (33.3%)
**Education**			
Illiterate	0	20 (16.3%)	69 (52.3%)
Primary school	1(0.8%)	92 (74.8%)	25 (18.9%)
High school or higher	0	11(8.9%)	38 (28.7%)
NA	121(99.1%)	0	0
**Professional status**			
Unemployed	0	18 (14.6%)	9 (6.8%)
Student	1(0.8%)	101(82.1%)	12 (9.1%)
Employed	0	4 (3.25%)	111(84.1%)
NA	121(99.1%)	0	0
**Household members**			
<5	55 (45.1%)	37 (30.1%)	70 (53%)
5 or more	67 (54.9%)	86 (69.9%)	62 (47%)
**Height (cm)**	90.1(11.9)	121.8 (18.3)	162.8 (7.9)
**Weight (Kg)**	12.2 (3.1)	22.6 (7.9)	59.9 (13.4)
**Haemoglobin level**	10.5 (1.7)	10.9 (1.3)	11.8 (1.8)
**Anaemia**			
Yes	56 (45.9%)	49 (37.1%)	40 (30.3%)
**Temperature (°c)**	36.3 (0.6)	36.5 (0.5)	36.3 (0.6)
**Consumption of tisane**			
Yes	13 (10.6%)	11 (8.9%)	28 (21.2%)
**Use of bednets**			
Yes	111(90.9%)	105 (85.3%)	105 (79.5%)
**Numbers of bednets**			
1	69 (56.5%)	71(57.7%)	84 (63.6%)
2	40 (32.8%)	35 (28.4%°	33 (25%)
3 or more	13 (10.6%)	17 (13.8%)	15 (11.4%)
**Use of bednets night before**			
Yes	106 (86.8%)	86 (69.9%)	88 (66.6%)

NA: not applicable

**Fig 3:**
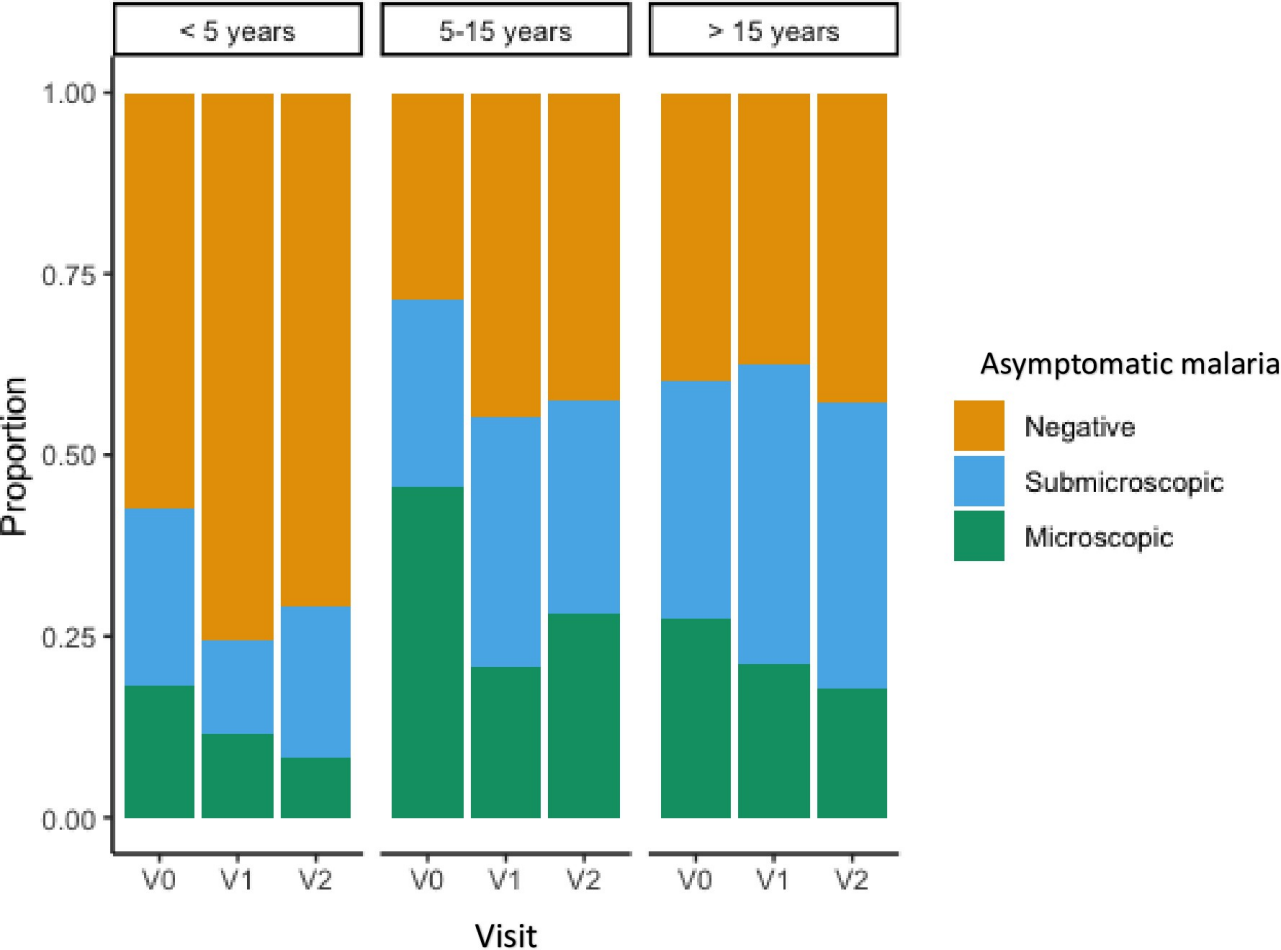
Distribution of asymptomatic malaria infection by age group and visit. V0: Inclusion; V1: Visit 2, V2: Visit 3.

Fig 3 illustrates the proportion of asymptomatic infectious reservoir observed over the three study visits and shows that participants aged 5–15 years and those over 15 years were the groups with the highest asymptomatic infection reservoirs. Around three-quarters of 5–15-year-olds were infected at the time of inclusion. Children under 5 were predominantly negative at all three visits. A high proportion of submicroscopic infections were found in all age groups, particularly in children aged 5–15 years old.

In the final multivariable model, the factors contributing to malaria infection status (submicroscopic and microscopic) dynamics were age between 5 and 15 years old (P < .001), over 15 years old (P < .001), Gender (Male) (P =. 017) and month of visit (Table 2).

**Table 2:** Ordinal polytomous mixed model (univariable and multivariable analysis).

	Malaria infection (N = 911)			
		OR (CI 95%)	AOR (CI 95%)	p-value adjusted
**Age (Ref : less than 5 years)**	5–15 years	4 .12 [2.55–6.67]	4.44 [2.67–7.37]	<0.001
	More than 15 years	2.80 [1.73–4.54]	3.67 [2.22–6.1]	<0.001
**Gender (Ref : Female)**	Male	1.63 [1.07–2.48]	1.62 [1.1–2.4]	0.017
**Consumption of tisane (Ref : No)**	Yes	1.50[0.87–2.59]		
**Use of bednets (Ref : No)**	Yes	0.67[0.41–1.10]	0.73 [0.45–1.2]	0.2
**Use of bednets night before (Ref : No)**	Yes	0.73 [0.45–1.20]		
**Month of visit (Ref : 1)**	2	0.46 [1.33–0.65]	0.50 [0.36–0.70]	<0.001
	3 or more	0,54[0,38–0,76]	0.59[0.42–0.85]	0.004

AOR: adjusted odds ratio; OR: odds ratio

**Fig 4:**
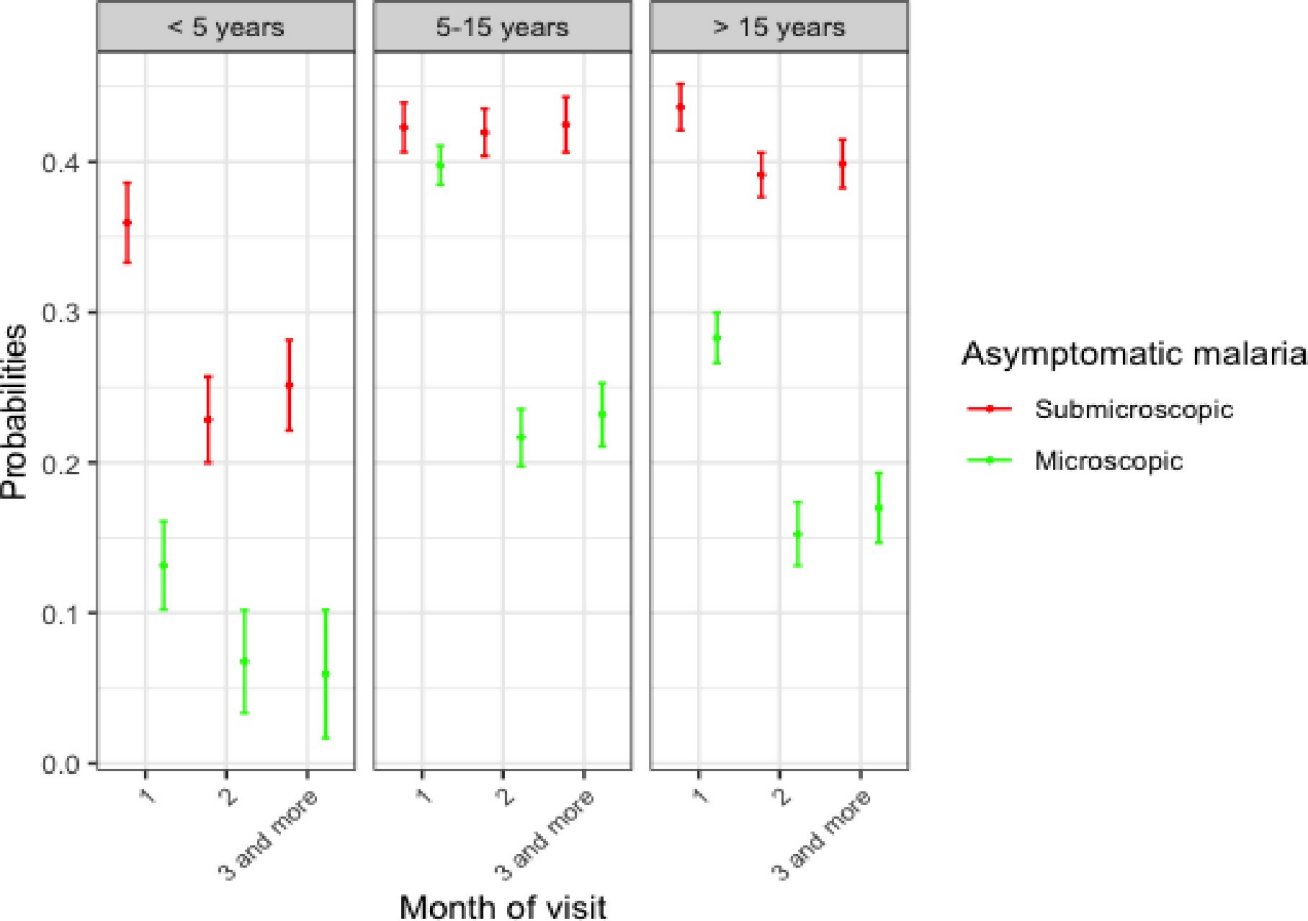
Mean risk of malaria infection by age group and month of visit. 1: August; 2: September; 3 and more: October and November.

The adjusted predicted probabilities of malaria infection according to age group and month of visit are shown Fig 4. The highest risk was found in children between 5 and 15 years old, and this risk was higher in the first month compared to the second and third month of follow-up. The risk of submicroscopic infections was higher than microscopic infections for all age groups at each visit.

**Fig 5:**
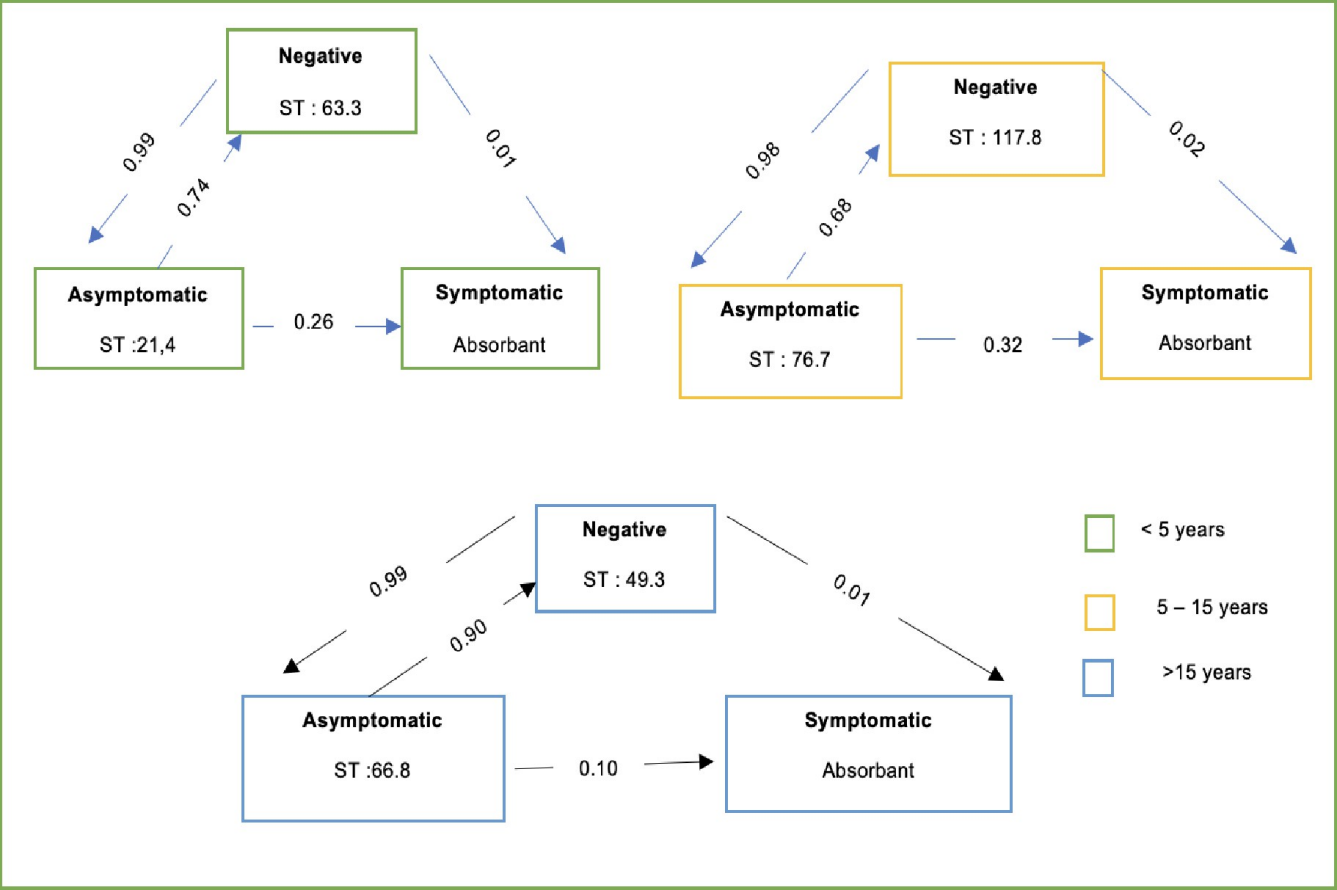
Estimated probabilities that each state is next and mean sojourn times. ST: sojourn times.

Fig 5 shows the results of the multi-state model with age group as a covariate. It shows that children aged 5 to 15 have the highest estimated sojourn time in the negative state (around 4 months), while estimated sojourn time in the other groups was around 2 months. Children under 15 were estimated to be 2 to 3 times higher risk of becoming symptomatic than individuals over 15.

## Discussion

The dynamics of asymptomatic malaria infections and its associated factors in facies of high perennial and seasonal transmission (south Benin) were analyzed, and the results showed a high and persistent prevalence of the asymptomatic malaria reservoir during the follow-up. Children aged 5–15 represented the most important reservoir, and pre-school and school-age children with asymptomatic infection were two to three times more at risk of becoming symptomatic than individuals aged over 15.

Malaria prevalence is mainly estimated based on microscopy, as many cases are identified in healthcare facilities, with microscopy remaining the standard method of diagnosis [27]. In our study, PCR detected a substantially higher number of asymptomatic infections than microscopy. The overall prevalence of malaria in our study was high, 30.4% (289/952) of infections were submicroscopic and 23.4% (223/952) microscopic, illustrating how traditional diagnosis methods underestimate asymptomatic cases, as reported by several asymptomatic malaria studies [28–30]. Asymptomatic parasite carriers constituted over 90% (471/512) of the human malaria reservoir in our setting, characterized by high seasonal and perennial transmission. The high rate of asymptomatic parasitaemia serving as reservoirs of infection may threaten any malaria control program in general, and the current malaria control program in Benin. This high rate must be considered in the evaluation of the global malaria control strategy. These findings underline the crucial importance of developing alternative high-sensitivity diagnosis (approaching at best the PCR sensitivity) deployable on the field for detecting most of asymptomatic malaria infections that might otherwise be missed by conventional methods. In addition, the implementation of targeted intervention programs, such as sustained follow-up, efficient mass screening to identify asymptomatic carriers, treatment campaigns, improved environmental conditions and control measures, could have significant impact. No significant asymptomatic malaria risk reduction was observed in net ownership (OR: 0.73, 95% CI: 0.45 to 1.2, p = 0.2). That may be partially explained by the fact that the (declarative) possession of mosquito nets does not automatically guarantee their effective use.

Participants aged 5 to 15 had the highest prevalence of asymptomatic malaria infection. Among them, 62.5% (190/304) were asymptomatic, among whom 47.4% (90/190) had submicroscopic infection. Our multivariate polytomous regression model demonstrated a strong association between malaria infection and age group. Precisely compared to children under 5 years of age, children aged 5 to 15 as well as individuals aged over 15 had a 3 to 4 higher risk of infection. These results are in line with a repeated cross-sectional study in Malawi which showed that over 88% of infected individuals were asymptomatic, particularly among school-age children between 6 and 15 and adults [12], with a higher risk of asymptomatic infection than younger children. A longitudinal study between 2017 and 2019 in Uganda demonstrated also through routine monitoring every 4 weeks for 2 years that the 5–15 age group contributed 59% to the infectious reservoir [15].

School-age children (between 5 and 15) appear to constitute a major malaria reservoir with high transmission potential. Those results from different epidemiological contexts are important, especially considering that school-aged often less exposed to antimalarial interventions. A literature review suggests that children in this age group are less inclined to use mosquito bed nets, which may provide a first explanation for these results [31].

The present study stands out, as far as we know, for its particular focus on estimating the average duration of asymptomatic infections within different age groups. The mean duration of asymptomatic infections was highest in children aged 5 to 15, closely followed by people over 15, probably due to a higher malaria immunity [11, 23, 32, 33] in the over 15 age group. The mean duration was substantially shorter for children under 5, more prone to develop symptomatic malaria according to our data.

Previous studies have examined the persistence of the asymptomatic reservoir, for example in Gambia [34]. A cohort of 42 individuals carrying *P*. *falciparum* infections, confirmed by PCR at the end of the transmission season was followed every month until the end of the dry season, to assess the persistence of their infections. Of them, 52% had 3 to 6 months persistent infections, with prominent roles of infections in children and submicroscopic infections [34].

This implies a potential longer contribution to parasite transmission of age groups above 5 years, which, added to the higher prevalence of asymptomatic infections, confirms the importance of the infectious reservoir in these age groups. In addition, school-age children (5–15) with asymptomatic malaria infection have shown a much higher risk of developing symptomatic malaria than people over 15. In people over 15, on average 90% clear the infection and become negative again.

All these results are crucial for fine-tuning malaria control and prevention strategies. The Malawi study recommended inclusion of school-age children in malaria surveillance and control programs, as well as the introduction of new interventions specifically for this age group [12]. Further longitudinal studies are needed, but our results extended those findings to different epidemiological settings and contribute to support these recommendations.

We observed that the cumulated prevalence of infection in children under 5 years of age was lower than that in other age groups (96/288 (33.3%)). However, children under 5 with asymptomatic infection were twice at risk to be symptomatically infected compared to children over 15. This result is also of high importance, knowing this age-group represents about 95% of malaria-related deaths [1, 35].

Male participants had a higher risk of being infected than female (OR: 1.62; 95% CI: 1.1 to 2.4). A study carried out in Senegal between 2002 and 2003 also revealed that males had a higher parasite density than females during the rainy season [36]. This difference in risk could be attributed to different behaviors and habits between genders [10]. It may be interesting to assess systematically the relation between gender and malaria exposure or infection, to consider possible gender specificities when implementing malaria prevention and control measures.

Our study has some limitations. First, the follow-up of our (pilot) study was short (<3 months). Consequently, the estimates of infection times could be more precise with a longer observation period. Secondly, the data collection on bed net variables were based on participants’ self-reports, which may lead to reporting biases. Thirdly, the time of entry into the initial states are left-censored, which may have slightly biased the estimation of sojourn times. Finally, the relatively limited sample size did not allow us to create a 4-state model distinguishing microscopic from submicroscopic infections, which would have been interesting. Despite these limitations, it did provide new and informative results.

## Conclusion

This longitudinal study in south Benin improved the knowledge of the dynamics of asymptomatic malaria infections in facies of high seasonal perennial transmission and identified some factors influencing these dynamics. Achieving malaria elimination targets depends on a thorough comprehension of this asymptomatic reservoir. Age appears as a key factor, with the prevalence of asymptomatic carriage being substantially higher in school-age children (5–15 years old). Given that this age group probably represents a significant proportion of the parasite reservoir, malaria control interventions targeting this age group should be recommended.
